# Antibiotic prophylaxis in penetrating traumatic brain injury: analysis of a single-center series and systematic review of the literature

**DOI:** 10.1007/s00701-022-05432-2

**Published:** 2022-12-19

**Authors:** Arjun Ganga, Owen P. Leary, Rahul A. Sastry, Wael F. Asaad, Konstantina A. Svokos, Adetokunbo A. Oyelese, Leonard A. Mermel

**Affiliations:** 1 Department of Neurosurgery, Warren Alpert School of Medicine, Brown University, Providence, RI 02903 USA; 2 Norman Prince Neuroscience Institute, Rhode Island Hospital, Providence, RI 02903 USA; 3 Department of Epidemiology and Infection Control, Lifespan Hospital System, Providence, RI 02903 USA; 4 Division of Infectious Diseases, Department of Medicine, Warren Alpert School of Medicine, Brown University, 593 Eddy St, Providence, RI 02903 USA

**Keywords:** Traumatic brain injury, CNS infection, Gun violence, Penetrating trauma

## Abstract

**Purpose:**

Penetrating traumatic brain injury (pTBI) is an acute medical emergency with a high rate of mortality. Patients with survivable injuries face a risk of infection stemming from foreign body transgression into the central nervous system (CNS). There is controversy regarding the utility of antimicrobial prophylaxis in managing such patients, and if so, which antimicrobial agent(s) to use.

**Methods:**

We reviewed patients with pTBI at our institution and performed a PRISMA systematic review to assess the impact of prophylactic antibiotics on reducing risk of CNS infection.

**Results:**

We identified 21 local patients and 327 cases in the literature. In our local series, 17 local patients received prophylactic antibiotics; four did not. Overall, five of these patients (24%) developed a CNS infection (four and one case of intraparenchymal brain abscess and meningitis, respectively). All four patients who did not receive prophylactic antibiotics developed an infection (three with CNS infections; one superficial wound infection) compared to two of 17 (12%) patients who did receive prophylactic antibiotics. Of the 327 pTBI cases reported in the literature, 216 (66%) received prophylactic antibiotics. Thirty-eight (17%) patients who received antibiotics developed a CNS infection compared to 21 (19%) who did not receive antibiotics (*p* = 0.76).

**Conclusions:**

Although our review of the literature did not reveal any benefit, our institutional series suggested that patients with pTBI may benefit from prophylactic antibiotics. We propose a short antibiotic course with a regimen specific to cases with and without the presence of organic debris.

## Introduction

In 2010, it was estimated that traumatic brain injury (TBI) led to approximately 2.2 million emergency department (ED) visits, 280,000 hospitalizations, and 50,000 deaths in the USA [4]. Penetrating traumatic brain injury (pTBI) is a severe type of TBI with a high rate of mortality [25]. Due to increasing fire arm violence and suicide attempts in the USA, with the firearm homicide rate increasing in 2020 to its highest level since 1994, more research is needed to address best practice in the management of pTBI patients [11, 21, 33]. Many pTBIs are a result of high-velocity ballistic trauma and such patients brought to an ED alive often expire within 24 h [8, 22, 23]. However, for those who have survivable injuries, providers are faced with unique challenges stemming from retained foreign bodies, tissue maceration, and burns due to thermal energy transfer from a projectile. Patients may also face a risk of infection stemming from foreign object entry into the brain parenchyma [27]. Rates of infection among pTBI patients were reported to be as high as 59% during the pre-antibiotic era of World War I [30]. However, studies conducted in the 1970s–1990s found the risk of infection to be between 4 and 11% [27]. The lower rates of infection reflect the advent of antibiotics and advances in operative and postoperative management [13]. We aimed to synthesize the most up-to-date information in the peer-reviewed literature to better understand the risk and prevention of central nervous system (CNS) infections following pTBI.

Prophylactic antibiotics are sometimes administered upon presentation, but high-quality evidence proving their benefit is lacking. In 1998, the International Brain Injury Association, the Brain Injury Association, the American Association of Neurological Surgeons, and the Congress of Neurological Surgeons collaborated on a set of guidelines for treating pTBI and recommended broad-spectrum antibiotics without details regarding which antibiotic to administer, nor the duration of antibiotic used in such cases [10, 34]. However, more recent publications noted insufficient evidence concerning the benefit of prophylactic antibiotics in decreasing CNS infections among pTBI patients [7, 14, 27]. Thus, we retrospectively evaluated consecutive patients with pTBI in a single institutional cohort to assess the relationship between empiric antibiotic administration and the development of clinical and microbiological evidence of CNS infection. We also performed a systematic review of pTBI cases to assess the impact of prophylactic antibiotic administration. We hypothesized that there would be no differences in CNS infection rates between those who did and did not receive prophylactic antibiotics.

## Materials and methods

### Literature review

The “Preferred Reporting Items for Systematic Reviews and Meta-Analyses” (PRISMA) Guidelines were followed [17].

### Search strategy and selection criteria

Two investigators (AG, OL) performed a literature review and independently selected articles for inclusion. All literature published before August 10, 2022, from three databases (PubMed, Embase, and Cochrane) was searched. Search terms for each database were as follows:

PubMed:

*(penetrating craniocerebral gunshot wounds [Title/Abstract] OR ptbi[Title/Abstract] OR penetrative TBI[Title/Abstract] OR penetrating brain injury[Title/Abstract] OR penetrating head trauma[Title/Abstract]) AND (antibiotics [Title/Abstract] OR prophylaxis [Title/Abstract] OR prophylactic [Title/Abstract] OR antibiosis [Title/Abstract] OR antibiotic[Title/Abstract]).*

Embase:

*('penetrating craniocerebral gunshot wounds':ab,ti OR 'ptbi':ab,ti OR 'penetrative tbi':ab,ti OR 'penetrating brain injury':ab,ti OR 'penetrating head trauma':ab,ti) AND ('antibiotics':ab,ti OR 'prophylaxis':ab,ti OR 'prophylactic':ab,ti OR 'antibiosis':ab,ti OR 'antibiotic':ab,ti).*

Cochrane:

*(“penetrating craniocerebral gunshot wounds” OR “ptbi” OR “penetrative TBI” OR “penetrating brain injury” OR “penetrating head trauma”) AND (“antibiotics” OR “prophylaxis” OR “prophylactic” OR “antibiosis” OR “antibiotic”).*

Screening of the articles was performed using Covidence (Covidence systematic review software, Veritas Health Innovation, Melbourne, Australia). The title and abstract of each study were independently screened by the two reviewers (AG, OL). Full texts of the retained studies were then screened independently by the same reviewers and issues were resolved by discussion with a third reviewer (RS). Inclusion criteria are detailed below.

### Inclusion criteria

- Published manuscript in a peer-reviewed scientific journal
- Full-text able to be located
- Published in English
- Presented new cases (i.e., not a meta-analysis or systematic review)
- Patients had an injury in which there was breach of the brain parenchyma by a foreign object
- It was able to be clearly discerned whether patients were given prophylactic antibiotics or not

### Risk of bias assessment

Bias was assessed independently by each reviewer using Cochrane Collaboration’s tool for assessing bias in non-randomized studies (ROBINS-I) [35]. Bias was evaluated across the following domains: bias due to confounding, bias in selection of participants into the study, bias in the classification of interventions, bias due to deviations from intended interventions, bias due to missing data, bias in measurement of outcomes, and bias in selection of the reported result. Evaluation using each domain was used to grade each study as having a low risk of bias, a moderate risk of bias, a serious risk of bias, a critical risk of bias, or no information. If two different bias ratings were assigned by each reviewer, the reviewers met and reached agreement on a single bias rating. A third reviewer was consulted if necessary.

### Data abstraction and synthesis

Data were abstracted by each reviewer independently onto standard data extraction forms. If an issue arose, reviewers met to find consensus regarding any difference in data extraction and/or interpretation in each study. The following data were extracted from published studies: number of patients studied; number of patients who did and did not receive prophylactic antibiotics; type of antibiotics administered if available; duration of antibiotics; number of CNS infections among the group who did and did not receive prophylactic antibiotics;.

All data processing and visualization was performed using Microsoft Excel (version 2016; Microsoft, Redmond, WA, USA) and Stata SE (StataCorp. 2017. *Stata Statistical Software: Release 15*. College Station, TX: StataCorp LLC.). The number and type of CNS infections were the primary outcome variables collected and were described as proportions. The analysis of our pooled patient sample from the literature involved calculating the pooled percentage of infections among those who did and did not receive prophylactic antibiotics. Next, we compared the proportion of CNS infections in the two groups using Fisher’s exact test for categorical data with an alpha-level of 0.05.

### Retrospective cohort

We performed a single-center, retrospective review of patients who received treatment for pTBI at Rhode Island Hospital from 2015 through 2019. Rhode Island Hospital is a tertiary care, Level I trauma center. Electronic records for all patients from the Trauma Patient Registry at Rhode Island Hospital were reviewed. Patient age, gender, and the mechanism of each injury were collected. In addition, we also collected information on the path of any  projectiles and the clinical management and laceration management of each case. Following presentation to the ED until discharge, each progress note in the electronic health record was scrutinized to collect all forms of CNS clinical management for each patients' injury (both medical and surgical), in addition to significant wound care events such as laceration repair, incision and drainage, etc. Additionally, the Glasgow Coma Scale (GCS) score at presentation was collected for each patient. Patients were excluded if they expired within 48 h of presentation, including if they expired on arrival to the ED, or if the injury did not involve a breach of the brain parenchyma. The type and duration of prophylactic antibiotics administered were recorded. Prophylactic antibiotic use was defined by at least one administered dose of intravenous antibiotics within 24 h of presentation to the ED. The primary outcome of the case series was the development of a CNS infection. Secondary outcomes included the management of a CNS infection, and if present, identification of any microorganisms that grew in cultures. Infection was identified based on either a positive culture following clinical suspicion or imaging consistent with a brain abscess on MRI. This study was approved by the Rhode Island Hospital/Lifespan Institutional Review Board.

## Results

We identified 23 patients who presented to our institution with pTBI. Two patients were excluded due to death within 48 h. Our final cohort consisted of 21 patients (20 male, 1 female; mean age 32 ± 13 years; median age 25 years). Fifteen patients (71%) experienced gunshot wounds to the head. Other mechanisms of injury included nail gun discharges to the head, as well as low-velocity projectiles. Two patients in our cohort died 2.5 days after presentation and a third patient died 7 days after presentation (Table 1).

**Table 1:** Patient demographic and clinical data

#	Age	Mechanism of inj	Trajectory of object	ED GCS	CNS/laceration management	Prophylactic systemic antibiotics	Course	Outcome	CNS Infection	Management	Cultured organism if any
1	34	Axe to head	Cranial, no path through sinus/face	14	Craniotomy for depressed skull fracture, evacuation of EDH	Cefazolin	ED, peri-operative	Survived	No		
2	46	Screwdriver to head	Cranial, no path through sinus/face	15	Bedside removal of screwdriver	None		Survived	Yes — intracranial Abscess (14 days post-admission)	Operative evacuation of abscess and removal of residual foreign body	*MSSA* *Finegoldia magna*
3	19	GSW to head	Cranial, no path through sinus/face	15	Craniotomy for foreign body removal	Cefazolin	Peri-operative only	Survived	No		
4	32	GSW to head	Cranial, no path through sinus/face	3	None	Cefazolin	ED	Died, 2.5 days after admission	No		
5	41	GSW to head	Cranial, no path through sinus/face	15	Wound incision and drainage	None		Survived	No — possible superficial wound infection	Subcutaneous fluid collection aspirated with resolution of concern	*MSSA*
6	25	GSW to head	Path through orbital wall, ethmoid sinus, and maxillary sinus	3	Craniotomy for GSW, temporal lobectomy, ventriculostomy placement	Cefazolin	Peri-operative only	Survived	No		
7	25	GSW to head	Path through infraorbital region, frontal, ethmoid, and maxillary sinuses	15	None	None	None	Survived	Yes — intracranial abscess (6 days post-admission) and pre-septal cellulitis	Operative evacuation of intracerebral abscess. Treated with incision and debridement by ophthalmology, meropenem for 6 weeks	*Clostridium perfringens, gram-negative rod (did not speciate)*
8	19	GSW to head	Cranial, no path through sinus/face	15	Laceration repair at bedside	None		Survived	Yes — intracranial abscess (10 days post-admission)	Managed non-operatively, treated with vancomycin/cefepime/metronidazole for 6 weeks	None
9	21	GSW to head	Path through frontal sinus	15	Craniotomy for debridement, elevation of skull fracture, sealing of frontal sinus, and titanium mesh cranioplasty	Vancomycin, ceftriaxone, metronidazole	Peri-operatively and post-operatively (2 days)	Survived	No		
10	21	GSW to head	Cranial, no path through sinus/face	4	None	Vancomycin, ceftriaxone, metronidazole	ED, inpatient (3 days)	Died, 2.5 days after admission	No		
11	21	GSW to head	Cranial, no path through sinus/face	15	None	Cefepime metronidazole, vancomycin. Developed allergic reaction, regimen changed to linezolid, aztreonam, metronidazole	ED, in patient (2 weeks)	Survived	No		
12	60	GSW to head	Path through left face with extensive soft tissue, osseous, frontal and sphenoid sinus involvement	12	None	Vancomycin, cefepime, metronidazole regimen. Transitioned to piperacillin and then amoxicillin	ED, in patient > 30 days	Survived	No		
13	54	GSW to head	Extensive hemifacial soft tissue and osseous injury, maxillary, ethmoid, and sphenoid sinus involvement	11	None	Cefazolin, cefalexin	ED, in patient (2 weeks)	Survived	No		
14	25	GSW to head	Cranial, no path through sinus/face	3	None	Vancomycin, ceftriaxone, metronidazole	ED, in patient (5 days)	Died, 7 days after admission	No		
15	53	Nail gun to head	Cranial, no path through sinus/face	8	EVD	Cefazolin, ceftriaxone, vancomycin	ED, in patient (5 days)	Survived	Yes, meningitis (31 days after admission)	Nafcillin/rifampin for 2-weeks, trimethoprim/sulfamethoxazole indefinitely for retained foreign-body	*Coagulase-negative staphylococci (CoNS)*
16	28	Nail gun to head	Cranial, no path through sinus/face	15	Craniotomy for nail removal	Vancomycin, cefepime, metronidazole	ED, peri-operative	Survived	No		
17	17	GSW to head	Cranial, no path through sinus/face	15	None	Cefazolin, vancomycin, metronidazole, ceftriaxone. Transitioned to clindamycin and ciprofloxacin	ED, in patient (10 days)	Survived	No		
18	23	Flare gun to head	Cranial, no path through sinus/face	15	None	Cefazolin	ED	Survived	No		
19	45	GSW to head	Cranial, no path through sinus/face	13	Laceration repair	Cefazolin	ED, in patient (5 days)	Survived	Yes, wound infection (7 days after admission) and subsequent brain abscess	Vancomycin/ceftriaxone/meropenem following identification of wound infection and craniotomy for abscess drainage	*MRSA*, *Enterobacter cloacae*
20	23	GSW to head	Cranial, no path through sinus/face	15	Cranial wound washout, craniotomy for elevation of depressed skull fracture, laceration repair	Cefazolin	ED, peri-operative	Survived	No		
21	44	Pellet gun to head	Cranial, no path through sinus/face	15	None	Vancomycin, metronidazole, ceftriaxone	ED, in patient (5 days)	Survived	No		

Seventeen local patients received prophylactic antibiotics (81%) as follows: seven (41%) received cefazolin monotherapy. Ten patients (59%) received different regimens of broad-spectrum antibiotics, most commonly including vancomycin, ceftriaxone, and metronidazole. Patients received antibiotics for varying durations, from 1 day to greater than 30 days. All antibiotic regimens were started within 24 h of presentation to our ED. Five patients (24%) developed CNS infections. Four had an intraparenchymal brain abscess and one had meningitis. Two of these patients required a surgical intervention. Brain abscesses were identified days 6, 7, 10, and 14 following admissions in the four patients. The patient with meningitis had symptom onset 31 days after admission. Three of four patients (75%) who did not receive prophylactic antibiotics developed a CNS infection compared to two of 17 (12%) who received prophylactic antibiotics (*p* = 0.03). Of the two patients who received prophylactic antibiotics and developed a subsequent infection, one patient received cefazolin alone (5-day regimen). The other patient first received cefazolin followed by ceftriaxone and vancomycin (5-day regimen). Four of the five patients who developed intracranial infections had routine bacterial cultures. The four patient’s cultures revealed growth as follows: *Finegoldia magna (F. magna) and methicillin-resistant Staphylococcus aureus (MRSA) (patient 1); MSSA (patient 2); Clostridium perfringens (C. perfringens) and unspecified gram-negative rods (patient 3); and coagulase-negative staphylococci (CoNS) (patient 4)*. One of the two patients who received prophylactic antibiotics developed an infection despite appropriate antibiotic coverage for the organisms that grew in culture. Of note, the other patient in the group who did not receive prophylactic antibiotics developed a superficial wound infection. Thus, all four patients who did not receive prophylactic antibiotics had either a CNS or non-CNS localized infection.

**Fig. 1:**
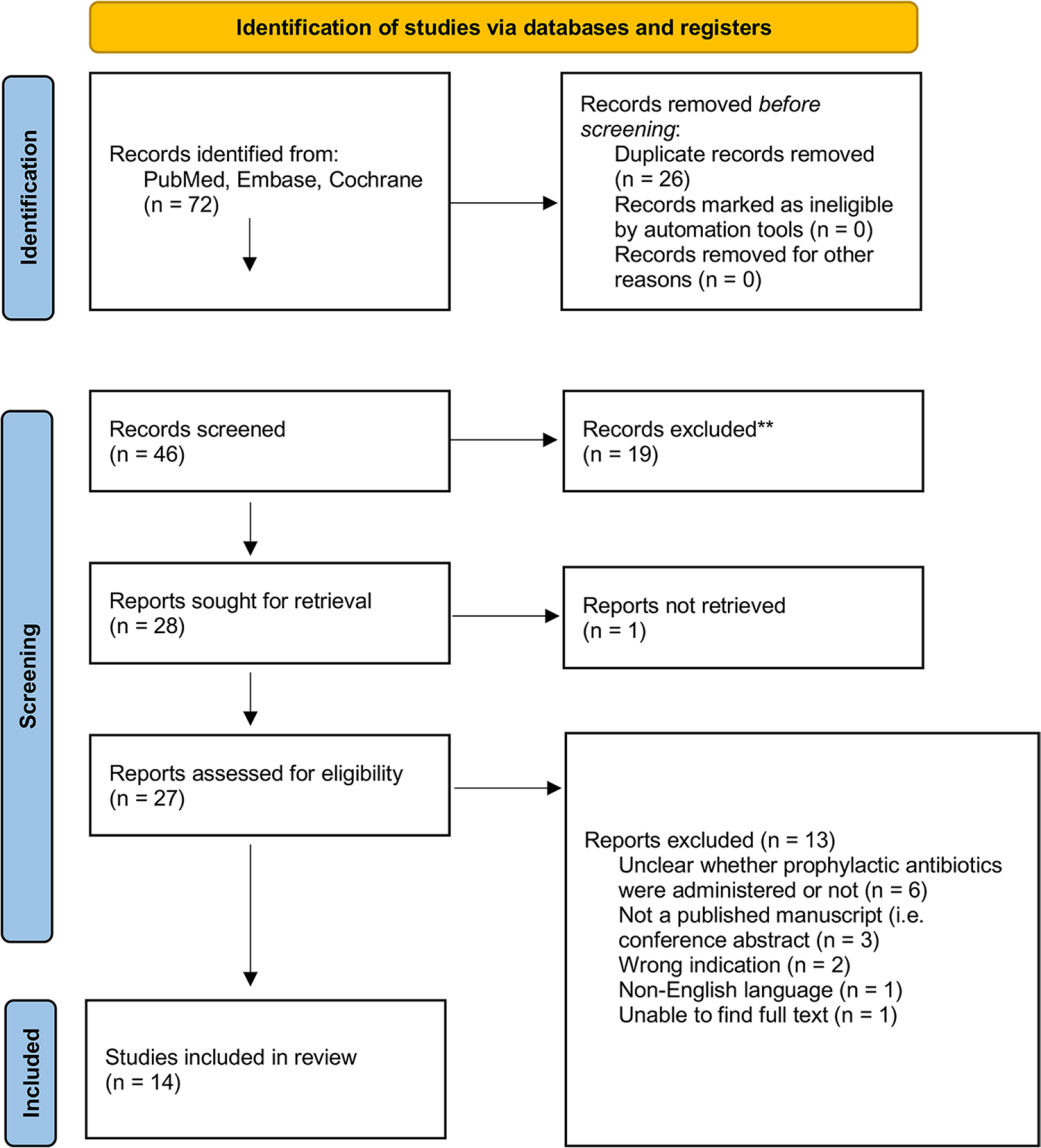
PRISMA flow diagram

Our literature search returned 72 studies (Fig. 1). Fourteen publications were incorporated in our analysis. A majority of the patients in our literature review came from three large case series [7, 14, 24], while the remainder consisted of patients from 11 case reports [1, 5, 12, 16, 18–20, 26, 29, 31, 32]. Thirteen studies were excluded most commonly because it was unclear if prophylactic antibiotics were administered (*n* = 6), the study was a conference presentation not published in a peer-reviewed journal (*n* = 3), the injury was not a penetrating injury involving a breach of the brain parenchyma (*n* = 2), the article was in a non-English language (*n* = 1), or the authors were unable to access the full text of the article (*n* = 1). The step-by-step process of the literature review is detailed in Fig. 1. We identified 327 unique pTBI cases; 216 (66%) received prophylactic antibiotics as a single agent or combination antibiotic regimen. The type and duration of antibiotics utilized varied widely. Among patients who received antibiotics, 38 (17%) developed an infection, compared to 21 who did not develop an infection (19%; *p* = 0.76, Table 2).

**Table 2:** Patients from the literature review and CNS infection outcomes

Author (year)	Journal	ROBINS-1 risk of bias	# of patients	# who received ppx abx	Type of abx	Duration of abx	# who did not receive ppx abx	Infections among ppx abx group and type	Infections among non-ppx abx group and type
Marut et al. 2020	Clin Neurol Neurosurg	Low	33	24/33	Mix of 1st–3rd generation cephalosporins, other beta-lactams, and non-beta-lactams	Median: 24 h (range: 7–84)	9/33	0/24	0/9
Xue et al. 2020	World J Clin Cases	Moderate	2	2/2	Patient 1: Vancomycin + meropenem Patient 2: Meropenem	N/a	0/2	0/2	N/a
Savitri et al. 2020	Int J Surg Case Rep	Moderate	1	1/1	Ceftriaxone	Single peri-operative dose	0/1	1/1, cranial abscess	N/a
Tabibkhooei et al. 2017	Neuroradiol J	Moderate	2	2/2	Broad-spectrum in both, not specified	N/a	0/2	1/2, bacterial meningitis	N/a
Jimenez et al. 2013	World Neurosurg	Low	160	59/160	Mostly 3^rd^ generation cephalosporins, also ampicillin/sulbactam	All administered for at least 72 h	101/160	20/59, mix of meningitis and cranial abscesses	20/101, mix of meningitis and cranial abscess
Okura et al. 2021	Trauma Case Rep	Moderate	1	1/1	Vancomycin and ceftriaxone	N/a	0/1	0/1	N/a
Samuthrat et al. 2017	World Neurosurg	Low	1	1/1	Meropenem, vancomycin, metronidazole	N/a	0/1	0/1	N/a
Ardill et al. 2003	Surg Neurol Aug	Moderate	1	1/1	Chloramphenicol, penicillin	N/a	0/1	0/1	N/a
Sirko et al. 2019	Georgian Med News	Moderate	121	121/121	Cefazolin	5 days	0/121	14/121, all intracranial purulent septic complications	N/a
Williams et al. 2014	Front Neurol	Low	1	1/1	Ceftriaxone, vancomycin, metronidazole	N/a	0/1	1/1, fever, cerebritis	N/a
Gutiérrez-González et al. 2008	Clin Neurol Neurosurg		1	1/1	Vancomycin, ceftazidime, metronidazole	7 days	0/1	0/1	N/a
Rammo et al. 2012	World Neurosurg	Low	1	1/1	Cefepime	7 days	0/1	1/1, bacterial meningitis	N/a
Vo et al. 2017	Cureus	Moderate	1	1/1	N/a	N/a	0/1	0/1	N/a
Lew et al. 1990	J Clin Microbiol	Low	1	0/1	None	N/a	1/1	N/a	1/1, abscess
Total:			327	216/327			111/327	38/216 (17.6%)	21/111 (18.9%)

## Discussion

The literature assessing outcomes following administration of prophylactic antibiotics to patients presenting with pTBI consists of case series. The two largest studies involve 160 [7] and 121 patients, respectively [24]. The former study followed patients with penetrating cerebral gunshot wounds caused by low-velocity projectiles admitted to a hospital in Medellin, Colombia. The authors reported an infection rate of 34% and 20% among patients who did and did not receive prophylactic antibiotics, respectively. All antibiotics were administered for at least 72 h. The pathogens most frequently identified were *MSSA* (54%), *Streptococcus pneumoniae* (15%), and *Klebsiella pneumoniae* (15%). This study demonstrated a higher infection rate in both groups when compared to our case series. The second study followed patients admitted to a neurosurgery service with penetrating gunshot wounds sustained in a local armed conflict in Ukraine [24]. Prophylactic antibiotics were administered using US military guidelines [6] consisting of 1gm of cefazolin every 8 h for 5 days. The authors reported 12% of their cohort developed an intracranial infection, similar to the findings of our case series. In another study involving pTBI at a large American urban hospital, 24 of 33 patients received prophylactic antibiotics; none of the 33 patients developed a CNS infection [14].

The decision to administer prophylactic antibiotics to pTBI patients is largely based on prior guidelines and neurosurgeon preference. One such guideline was borne out of a collaboration between the International Brain Injury Association, the Brain Injury Association, the American Association of Neurological Surgeons, and the Congress of Neurological Surgeons [34].This widely cited guideline recommends routine use of an unspecified regimen of broad-spectrum antibiotics for such cases. The British Society for Antimicrobial Chemotherapy performed a systematic review of both military and civilian penetrating craniocerebral injuries and despite expressing dissatisfaction with the available data, unspecified, broad-spectrum antibiotic prophylaxis was recommended for all military and civilian injuries [2, 27]. Recent guidelines from the Infectious Diseases Society of America (IDSA) do not contain any recommendations concerning prophylaxis for pTBI cases [28].

Many guidelines for pTBI management come from the military literature [3]. The United States Department of Defense Centers for Excellence for Trauma published guidelines recommending prophylactic antibiotics for penetrating injuries [15]. This guideline recommends cefazolin (2gm intravenously every 8 h) or clindamycin (600 mg intravenously every 8 h) for an unspecified duration. If the wound is visibly contaminated with organic debris, the guideline suggests the addition of metronidazole (500 mg, intravenously every 8 to 12 h). The United States Army Center for Surgical Research also recommends use of prophylactic antibiotics for penetrating head injury [6]. This group recommends cefazolin (1gm intravenously every 8 h) for 5 days, with an extended duration if there is gross contamination of the wound.

There is variability concerning use of prophylactic antibiotics based on a neurosurgeon's preference. A 1991 survey of American neurosurgeons [9] found that 87% of respondents generally use prophylactic antibiotics for pTBI cases; 24% administer antibiotics for 1–3 days, 19% for 4–5 days, and 68% for more than 5 days using cephalosporins (87%), chloramphenicol (24%), penicillin (16%), an aminoglycoside (12%), or vancomycin (6%), with fewer using either erythromycin, miconazole, or tetracycline. Of note, 49% of respondents reported using multiple antibiotics.

Our own review and case series does not unequivocally clarify the utility of prophylactic antibiotics for pTBI, reflecting a lack of randomized trials. However, based on available data, including the pathogens grown from intraoperative cultures in pTBI cases that went on to develop a CNS infection, we recommend using a short course of prophylactic antibiotics such as cefazolin 2gm, intravenously every 8 h; if organic debris is present, we instead recommend use of ceftriaxone 2gm every 12 h and metronidazole 7.5 mg/kg every 6 h along with debridement as clinically indicated. Of note, some pTBI cases resulting in a CNS infection may have been due to inadequate source control of infected brain tissue or bone, rather than antibiotic failure.

### Limitations

Our case series is limited by the small sample size. Additionally, we did not control for any patient-level characteristics that may have predisposed to developing an infection. Our results should be interpreted with caution given the degree of heterogeneity in our included sample. This heterogeneity stems from the varying injury mechanisms, the variety of projectiles, as well as their trajectories into the skull and local microbial flora in each case. These factors may have biased our results, changing the risk-to-benefit ratio regarding antibiotic administration at the patient-specific level, making it difficult to standardize an all-encompassing prophylactic regimen and/or definitely prove the benefit of prophylactic antibiotic use. Our literature review is also limited by a lack of any prospective, randomized studies. As such, our analysis and pooled proportions of CNS infections should be interpreted with caution. Furthermore, infections may develop years after penetrating brain injuries and we may have missed such infections in our case series and such outcomes may have been missed in published studies. 

## Conclusion

Prophylactic antibiotics are commonly used in pTBI cases. There is variability in the literature regarding their use, and when instituted, variability in the choice of antibiotics and their duration. We propose a short antibiotic course with a regimen specific to cases with and without the presence of organic debris. As rates of firearm violence and suicide attempts have risen in the USA, and due to the present military conflict in Ukraine, clear, evidence-based guidance is sorely needed [11]. The data analyzed in the current study are not publicaly available due to their personal nature but additional details may be available upon reasonable request.

## Data Availability

The data analyzed in the current study are not publicly available due to their personal nature, but additional details may be available upon reasonable request.

## Abbreviations

CNS: Central nervous system
ED: Emergency department
g: Gram
GCS: Glasgow Coma Scale
IDSA: Infectious Diseases Society of America
mg: Milligram
MRSA: Methicillin-resistant *Staphylococcus aureus*
MSSA: Methicillin-susceptible *Staphylococcus aureus*
pTBI: Penetrating traumatic brain injury
TBI: Traumatic brain injury
